# Antimicrobial Octapeptin C4 Analogues Active against Cryptococcus Species

**DOI:** 10.1128/AAC.00986-17

**Published:** 2018-01-25

**Authors:** Jessica L. Chitty, Mark S. Butler, Azzah Suboh, David J. Edwards, Matthew A. Cooper, James A. Fraser, Avril A. B. Robertson

**Affiliations:** a Australian Infectious Diseases Research Centre, School of Chemistry and Molecular Biosciences, The University of Queensland, St. Lucia, Queensland, Australia; b Institute for Molecular Bioscience, The University of Queensland, St. Lucia, Queensland, Australia

**Keywords:** Cryptococcus neoformans, octapeptin, antifungal agents, lipopeptide

## Abstract

Resistance to antimicrobials is a growing problem in both developed and developing countries. In nations where AIDS is most prevalent, the human fungal pathogen Cryptococcus neoformans is a significant contributor to mortality, and its growing resistance to current antifungals is an ever-expanding threat. We investigated octapeptin C4, from the cationic cyclic lipopeptide class of antimicrobials, as a potential new antifungal. Octapeptin C4 was a potent, selective inhibitor of this fungal pathogen with an MIC of 1.56 μg/ml. Further testing of octapeptin C4 against 40 clinical isolates of C. neoformans var. *grubii* or neoformans showed an MIC of 1.56 to 3.13 μg/ml, while 20 clinical isolates of C. neoformans var. *gattii* had an MIC of 0.78 to 12.5 μg/ml. In each case, the MIC values for octapeptin C4 were equivalent to, or better than, current antifungal drugs fluconazole and amphotericin B. The negatively charged polysaccharide capsule of C. neoformans influences the pathogen's sensitivity to octapeptin C4, whereas the degree of melanization had little effect. Testing synthetic octapeptin C4 derivatives provided insight into the structure activity relationships, revealing that the lipophilic amino acid moieties are more important to the activity than the cationic diaminobutyric acid groups. Octapeptins have promising potential for development as anticryptococcal therapeutic agents.

## INTRODUCTION

Despite dwindling interest in antimicrobial development from pharmaceutical companies, academia is continuing to pursue a pipeline of research in this area (1, 2). Much of this work has focused on pathogens that are a significant problem in the first world, particularly nosocomially acquired infections caused by Staphylococcus aureus and Enterobacteriaceae, in which drug resistance is a major concern (3). In contrast, important pathogens more prevalent in developing nations tend to be overlooked. One such pathogen is Cryptococcus neoformans, a basidiomycete yeast that is a leading cause of death in HIV/AIDS patients (4). Infection occurs in immunocompromised patients after airborne basidiospores or desiccated yeast cells are inhaled, ultimately entering the alveoli. The C. neoformans fungus can cause pneumonia or, more frequently, disseminate to the central nervous system to manifest as meningoencephalitis (5). In total, cryptococcal meningoencephalitis accounts for 15% of AIDS-related mortality (4). Although improved diagnosis, due to the development of a lateral flow assay, has enhanced the clinical outcomes of these patients, effective antifungal drugs are still a limiting factor in combating infection (6).

Treatment for cryptococcosis is reliant on multidrug therapy (7, 8). Current recommendations are induction therapy with amphotericin B and flucytosine, followed by consolidation and maintenance with fluconazole (9, 10). Fluconazole and amphotericin B exploit the presence of ergosterol in the C. neoformans cell membrane; fluconazole inhibits ergosterol biosynthesis, and amphotericin B binds ergosterol on the cell membrane to cause cell leakage (11, 12). Flucytosine is an antimetabolite prodrug which disrupts nucleic acid metabolism, thereby inhibiting both protein and DNA synthesis in fungi (13). Alarmingly, sustained use of these few antifungals has led to the emergence of resistant strains (14–21). Although resistance to all three drugs has been observed, cryptococcosis treatment has not significantly altered in over 2 decades despite unacceptably high mortality rates (7, 8). New therapeutics are urgently needed.

A resource-effective approach to combat C. neoformans is to capitalize on biophysical properties the fungus shares with other infecting microbes and repurpose drugs that are already in use treating these illnesses; this takes advantage of pharmacology and toxicity information already available. Given the interface between the host and infecting C. neoformans cells is the negatively charged polysaccharide capsule (22), agents whose mode of action is via interaction with negatively charged surfaces could potentially be exploited to treat cryptococcal meningoencephalitis. A series of such compounds, first discovered in the 1970s, are the octapeptins produced by Bacillus circulans (23–25). Octapeptins are cationic cyclic lipopeptides, with seven amino acids in the cyclic core and an eighth exocyclic amino acid linked to a lipid tail group (26–29). Despite early reports of activity against bacteria, fungi, and protozoa, octapeptins remain relatively unexplored, with surprisingly few investigations of this family of naturally occurring compounds (28, 30).

Other examples of cyclic lipopeptide molecules are polymyxin B (PMB) and polymyxin E (PME; also known as colistin), broad-spectrum lipodecapeptides used against Gram-negative bacterial infections (31). PMB and PME are secondary metabolites of Paenibacillus polymyxa, a Gram-positive aerobic bacteria abundant in most rhizospheric soils (30, 32). These are particularly relevant in the context of antibiotic resistance as PME, despite its nephrotoxicity, is used as a last line of defense against drug-resistant Gram-negative bacteria (33, 34). In these pathogens, the binding of PMB or PME occurs primarily via the polycationic cyclic heptapeptide core of the drug, displacing calcium and magnesium ion bridges in lipopolysaccharide, while the fatty acid of the linear peptide tail interacts with the membrane, leading to permeability changes and cell death (35, 36). PMB has weak activity against C. neoformans and more potent activity when used in synergy with azoles against this fungal pathogen (37, 38).

We report here the first detailed *in vitro* antifungal study of pure synthetic octapeptin C4 and direct comparative data to PMB and PME against a panel of fungal pathogens: Candida albicans, Candida glabrata, Candida parapsilosis, Cryptococcus gattii, Cryptococcus neoformans, and one pathogenic mold, Aspergillus fumigatus. Furthermore, nine octapeptin C4 analogs, constructed using solid-phase peptide synthesis, were used to examine structure-activity relationships (SAR). The activities of compounds were determined by broth microdilution assays, as well as time course assays to determine activity against capsule- and melanin-induced C. neoformans cells. Understanding the SAR of these compounds is the first step in guiding synthesis of more active and efficacious derivatives for the treatment of life-threatening disseminated fungal diseases.

## RESULTS

### Octapeptin C4 is 8-fold more potent than PMB at inhibiting growth of C. neoformans.

**FIG 1:**
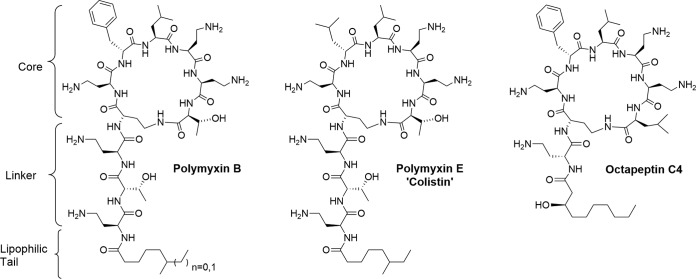
Cationic cyclic lipopeptide antibacterial agents. Polymyxin B and polymyxin E are produced by P. polymyxa. The related species B. circulans produces octapeptin C4.

A broth microdilution assay was initially used to characterize the antifungal activity of the cyclic lipopeptides (39, 40). Four common pathogenic yeasts (C. albicans, C. glabrata, C. parapsilosis, C. neoformans, and C. gattii) and one pathogenic mold (A. fumigatus) were tested to determine the MIC for each compound (Table 1) using broth microdilution. Similar to previous reports (37, 38), PMB exhibited specificity toward C. neoformans, with both strains tested showing an MIC of 12.5 μg/ml, whereas the C. gattii strain had an MIC of 25 μg/ml. In contrast, the growth of C. albicans, C. glabrata, C. parapsilosis, and A. fumigatus was not inhibited at the highest concentration of test compound (100 μg/ml) (37, 38). The antibiotic of last resort, PME, did not show significant antifungal activity against any of the species analyzed in this assay. The only structural difference between these molecules is the presence of a phenylalanine residue at position 6 in PMB; in PME a d-leucine is present at this location (Fig. 1).

**TABLE 1:** Antifungal MICs of PMB, PME, and octapeptin C4 as determined by broth microdilution assays against common fungal pathogens

Compound	MIC (μg/ml)							
	C. albicans	C. glabrata	C. parapsilosis	A. fumigatus	C. gattii	C. neoformans		
						H99	ATCC 90113	H99 (*cap59*Δ)
PMB	>100	>100	>100	>100	25.0	12.5	12.5	50
PME	>100	>100	>100	>100		100		
Octapeptin C4	>100	100	>100	>100	3.13	1.56	1.56	6.25

Analysis of the antifungal activity of octapeptin C4 revealed an MIC of 1.56 μg/ml against both C. neoformans strains and 3.13 μg/ml against C. gattii. As with PMB, the other fungal strains were resistant up to concentrations of 100 μg/ml or above. Octapeptin C4 is therefore not a broad-spectrum antifungal, but a specific inhibitor of Cryptococcus; compared to PMB, octapeptin C4 was 8-fold more potent. Clinical isolates of C. neoformans var. *grubii* and C. neoformans var. neoformans displayed MIC results between 1.56 to 3.13 μg/ml in 40 strains. A total of 20 clinical isolates of C. neoformans var. *gattii* displayed more variation in MIC results between 0.78 μg/ml and 12.5 μg/ml (see Table S1 in the supplemental material). In each case, octapeptin C4 was equivalent to, or more potent than, the current antifungal drugs fluconazole and amphotericin B, which were used as comparative controls.

### The C. neoformans capsule influences sensitivity to octapeptin C4.

The different susceptibility of C. neoformans and C. gattii to PMB compared to other pathogenic fungi such as C. albicans and A. fumigatus has been postulated to arise from differences in cell surface composition (38). One key difference is the presence of a negatively charged polysaccharide capsule in Cryptococcus spp. proposed to concentrate the cationic lipopeptides at the cell surface (38). To examine this potential interaction for the octapeptin series, a C. neoformans *cap59*Δ mutant strain deficient in the secretion of negatively charged glucuronoxylomannan (an α1–3-linked mannan containing β1,2 and β1,4 xylosyl substitutions, as well as β1,2-linked glucuronyl residues) (41, 42), was tested against octapeptin C4 in comparison to PMB. These data suggested the altered capsule of the *cap59*Δ mutant provides a degree of protection against cationic lipopeptides such as PMB and octapeptin C4.

**FIG 2:**
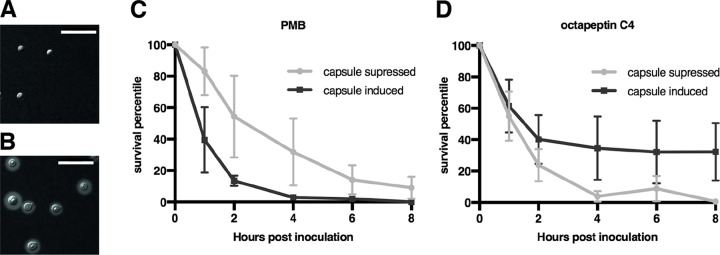
Capsule formation enhanced the fungicidal effect of PMB but retarded the fungicidal activity of octapeptin C4. (A) C. neoformans grown on capsule inducing RPMI 1640 agar medium (without l-glutamine and sodium bicarbonate) with incubation at 37°C in 5% CO_2_ for 3 days. Scale bar, 20 μm. (B) C. neoformans grown on capsule-suppressing media, YPD containing 1 M NaCl at 30°C in ambient air for 3 days. (C and D) Survival of capsule-induced or -suppressed C. neoformans cells after incubation with PMB at 12.5 μM (*P* = 0.0288) (C) or octapeptin C4 at 1.56 μM (*P* = 0.0186) (D). The data represent the means of two replicates, and the error bars represent the standard errors of the mean (SEM).

To further probe the effect of the quantity of wild-type, negatively charged polysaccharide capsule on octapeptin C4 sensitivity, time course cell viability assays were conducted (Fig. 2).

In the presence of PMB, it took only around 1 h to observe a 50% reduction in viability of the fungal cells with large capsule, whereas it took five times longer to observe an equivalent effect of the capsule suppressed fungal cells. In contrast, it was found that the reciprocal was true for the fungicidal activity of octapeptin C4; in this case, abundant capsule production led to a small but statistically significant increase in survival.

### C. neoformans melanization does not affect activity of octapeptin C4.

**FIG 3:**
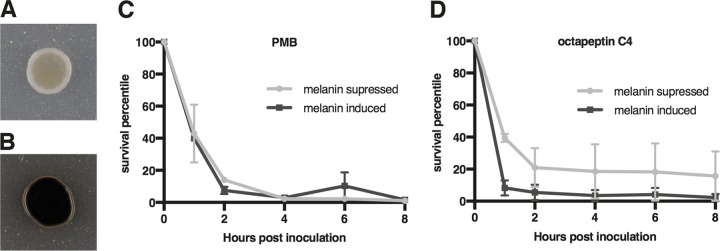
Octapeptin C4 fungicidal activity is not influenced by the melanization of C. neoformans. (A) Melanin suppressed C. neoformans. (B) Melanin induced C. neoformans. (C and D) Graphs show survival of melanized and nonmelanized C. neoformans cells after incubation with either PMB at 12.5 μM (no significance *P* = 0.9840) (C) or octapeptin C4 at 1.56 μM (*P* = 0.0143) (D). *, *P* < 0.01. Samples from each flask were taken 0, 1, 2, 4, 6, and 8 h after inoculation, spread onto a YPD plate, and incubated at 30°C for 3 days, at which time the colonies were counted. The data represent the means of two replicates, and the error bars represent the SEM.

The activity of PMB or octapeptins against melanized C. neoformans has not been previously reported. We used a time course cell viability assay where melanization was induced by growth on l-DOPA agar medium and suppressed by growth on YPD agar medium (Fig. 3). PMB showed no difference in activity relative to the degree of melanization, whereas the fungicidal activity of octapeptin C4 was slightly enhanced with heavily melanized C. neoformans cells.

SAR studies revealed that the lipophilic groups are more important than the diaminobutyric acid residues for the anticryptococcal activity of octapeptin C4. We also investigated the functional importance of key residues in octapeptin C4 to antifungal activity by performing an alanine scan, using a published route to synthesize a series of nine octapeptin C4 derivatives that each differ from the parent compound by a single residue converted to an alanine (43).

The structure-activity relationships of PMB, octapeptin C4, and the nine alanine scan analogs were explored using MIC assays with the four Cryptococcus strains used so far in the study (H99, ATCC 90113, MMRL 2651, and the *cap59*Δ mutant). The cationic nature of octapeptin C4 is a product of its four diaminobutyric acid (Dab) residues; if electrostatic interactions between these cationic Dab groups and the polysaccharide capsule were a key feature of the antifungal activity of octapeptin C4, then altering these to noncationic amino acids should lead to a reduction in efficacy against the Cryptococcus wild-type strains. The octapeptin C4 Dab residues were therefore sequentially replaced with alanine in compounds P1, P3, P6, and P7.

A consistent increase in the MIC values was observed from 1.56 to 6.5 μg/ml in the H99 and ATCC 90113 strains, indicating that the Dab residues were all equally important for the antifungal activity of the octapeptins (Table 2). Furthermore, maintaining the d configuration of the natural octapeptin structure at position 1 (P1d) gave a lower MIC than changing it to the l configuration (P1l). These results were mirrored in the MMRL 2651 C. gattii strain but with higher MIC values.

**TABLE 2:** MIC of octapeptin C4 and alanine scan derivatives

Compound	MICs (μg/ml) against various Cryptococcus strains^*a*^			
	H99	ATCC 90113	MMRL 2651	H99 (*cap59*Δ)
Octapeptin C4	1.56	1.56	3.13	6.25
P1L	12.5	12.5	12.5	25.0
P1D	6.25	6.25	12.5	12.5
P3	6.25	6.25	12.5	12.5
P4L	50.0	25.0	100	100.0
P4D	12.5	12.5	25.0	50.0
P5	12.5	12.5	25.0	50.0
P6	6.25	6.25	6.25	12.5
P7	6.25	6.25	12.5	12.5
P8	50.0	6.25	50.0	100

a Experiments were performed in triplicate; values were consistent across replicates.

Our SAR studies also probed the effect of the lipophilic moieties Phe (P4d and P4l) and Leu (P5 and P8) by sequentially switching to alanine. These changes showed that the lipophilic groups were potentially more important for antifungal activity than the cationic Dab residues since the MIC values were even poorer. The stereochemical configuration of the natural analog at position 4 is “d,” and maintaining this in the alanine variant (P4d) gave a better MIC than the l configuration (P4l). Overall, the nine alanine derivatives were all less active than the original octapeptin C4.

## DISCUSSION

To address the need for new antimicrobials, in particular against fungal pathogens, we can potentially repurpose classes of drugs that have already been studied and characterized against other pathogens by exploiting shared physiologies. PMB has been proposed to target the negatively charged surface of Gram-negative bacteria, a characteristic shared with C. neoformans. *In vitro* assays of PMB showed that a relatively high concentration of PMB is required to exert fungicidal effect, most likely precluding monotherapy due to associated toxicity at high doses; however, synergy with the azole class of antifungals suggests that it may be clinically useful in treating fungal disease (37, 38). Although there is striking similarity between the structures of polymyxins (PMB and PME) and the octapeptin class of antibiotics, octapeptins are functionally distinct as they retain activity against polymyxin-resistant bacteria (43). This led us to explore octapeptins as potential antifungal agents and to study which structural features were key to their antifungal activity.

Antifungal MICs determined in broth microdilution assay (Table 1) showed that octapeptin C4 exhibited a Cryptococcus-specific MIC of 1.56 μg/ml and a promising lead compound. Octapeptin C4 was ∼8-fold more potent than PMB, with an MIC of 12.5 μg/ml, and >60-fold more potent than PME. In keeping with these data, clinical isolates of C. neoformans var. *grubii* and C. neoformans var. neoformans displayed MIC results between 1.56 and 3.13 μg/ml in 40 strains. A total of 20 clinical isolates of C. neoformans var. *gattii* displayed more variation in MIC results between 0.78 and 12.5 μg/ml (see Table S1 in the supplemental material). Intriguingly, a phenylalanine residue is present at position 4 in octapeptin C4, a location equivalent to the phenylalanine residue key to the antifungal activity of PMB. In these MIC tests, the clinical compounds fluconazole and amphotericin B were used as comparative controls, octapeptin C4 was equivalent or more potent than either drug *in vitro* (see Table S1 in the supplemental material). The differences in membrane composition of bacterial cells versus eukaryotic fungal cells (high levels of sterols, neutral lipids, and low membrane potential) (44, 45) are likely responsible for the increased MIC in pathogenic fungi with PMB (37, 38). Octapeptin C4 showed a 50% cytotoxic concentration of 31 μM in the human kidney-2 cell line and a similar activity in hRPTEC cells based on a lactate dehydrogenase release assay (43). Although improvement in this cytotoxicity measure would be desirable, there is a therapeutic window, which may be improved through chemical modification and SAR studies.

Cryptococcus has a protective, negatively charged, polysaccharide layer of variable thickness which encapsulates the yeast cell. The capsule modulates, and provides protection from, immune responses of the host and promotes virulence (46, 47). The *cap59*Δ mutant of C. neoformans H99 is often described as acapsular and was used to further investigate the effect of capsule on PMB, octapeptin C4, and alanine scan analogues on antifungal activity. An increased resistance to PMB was found, in agreement with literature precedent (38), and was also true for the octapeptins. However, the *cap59*Δ strain is not truly acapsular; it can produce both glucuronoxylomannan and galactoxylomannan but only secretes galactoxylomannan to form its capsule. This altered capsule seems to provide a degree of resistance to penetration by the tested compounds. The capsule of the physiologically relevant native C. neoformans H99 strain has glucuronoxylomannan as the principal component, and this strain is more suited to prediction of capsule influence on drug efficacy. In agreement with literature precedent (38), the presence of this capsule increased susceptibility of C. neoformans to PMB, as shown by the incubation of fungal cells with capsule production induced (37, 38). In contrast, under the same conditions, octapeptin C4 antifungal activity was slightly suppressed. This may give an early indication of a divergent mode of action where octapeptin C4 activity cannot be fully explained by ionic interactions with the capsule.

A second key virulence factor of C. neoformans that could interact with cationic lipopeptides is melanin, a negatively charged pigment in the cell wall (48). Melanin granules anchored in the fungal cell wall form ordered concentric layers and are known to contribute to survival and pathogenesis (49). Melanization alters the susceptibility of C. neoformans to current antifungals such as amphotericin B and caspofungin as these bind to melanin, adversely affecting their antifungal activity and consequently the treatment of patients (50–52). However, other melanin binding agents have been reported to increase susceptibility of the fungal cell to the antifungal agents trifluoperazine and chloroquine (53); this is particularly significant for meningoencephalitis infections where melanization of C. neoformans is highly prevalent (48). Here, we observed that the antifungal activity of PMB and octapeptin C4 was not diminished against heavily melanized fungal cells; indeed, octapeptin C4 showed slightly enhanced activity. This was encouraging in considering these compounds as therapeutic agents since they are unlikely to show the altered susceptibility characteristic of amphotericin B and caspofungin.

Comparison of antifungal activity of polymyxin antibiotics to octapeptin C4 led us to explore the structural features of the octapeptins that played a key role in their activity. It is possible that the negatively charged capsule of Cryptococcus attracts and concentrates cationic lipopeptides increasing their antifungal effect, which is supported by the correlation of the number of positively charged Dab residues with decreased antifungal activity. Despite this apparent importance of the octapeptin Dab groups, it is noteworthy that PMB has five cationic Dab groups and yet is less effective against Cryptococcus than the octapeptin alanine scan variants with only three such residues. This further indicates that the charged interactions between the cationic Dabs and the negatively charged capsule do not entirely explain the mode of action for octapeptin C4.

The hypothesis that there is a correlation between Dab residues and a decreased antifungal activity is over simplistic; our SAR studies showed that lipophilic moieties of octapeptin C4 are even more important for the fungicidal activity than the Dab residues. Given that the polysaccharide capsule is highly hydrophilic and water accounts for 99% of the total volume and weight of the capsule (54), the lipophilic residues of octapeptin C4 are more likely to be critical in binding to the cell membrane.

**FIG 4:**
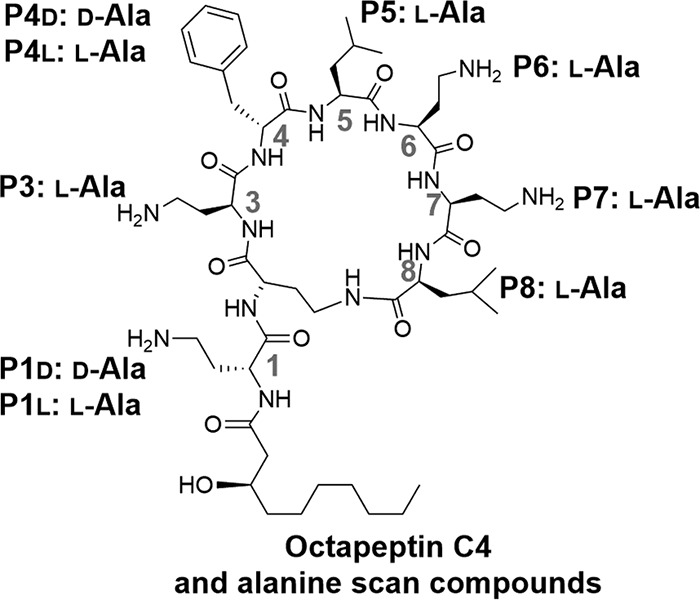
Nine octapeptin C4 alanine scan derivatives. All amino acids are l-isomers unless indicated otherwise.

The potency of octapeptin C4 as an antifungal agent against Cryptococcus laboratory strains and clinical isolates provides an exciting foundation on which to develop future therapeutics. Work is ongoing to investigate octapeptin C4 antifungal mode of action and activity *in vivo*. We are also studying other members of the octapeptin series and synthesizing analogues with enhanced activity/toxicity window (Fig. 4).

## MATERIALS AND METHODS

### Test compounds.

PMB sulfate, PME (colistin), fluconazole, and amphotericin B were purchased from Sigma-Aldrich (Australia). Octapeptin C4 and nine alanine scan derivatives were synthesized using a route reported previously (43). All compounds were solubilized in water, with the exception of amphotericin B, which was solubilized in dimethyl sulfoxide. For purity data, see the supplemental material.

### Strains and media.

Fungal strains were stored in 15% glycerol at −80°C until use. The fungal strains used were H99 (C. neoformans type strain) (55), *cap59*Δ (an acapsular H99 mutant) (56), ATCC 90113 (C. neoformans), MMRL 2651 (C. gattii), ATCC 90028 (Candida albicans), ATCC 90030 (Candida glabrata), ATCC 22019 (Candida parapsilosis), and ATCC MYA 3626 (Aspergillus fumigatus). Yeasts were cultured in liquid (1% yeast extract, 2% Bacto peptone, 2% glucose) or solid (additional 2% agar) yeast extract-peptone-dextrose (YPD) media at 30°C and maintained at 4°C for no longer than 2 weeks. A. fumigatus was prepared on potato dextrose medium at 30°C and maintained at 4°C for no longer than 2 weeks.

### *In vitro* assay of drug efficacy.

The MICs of the yeast strains for PMB, PME, and octapeptin C4 and its derivatives were determined in accordance with CLSI M27-A2 guidelines using YNB (Becton Dickinson, USA) medium supplemented with 10 mM ammonium sulfate and 2% glucose, a final inoculum concentration of 1.5 × 10^3^ to 2.0 × 10^3^ cells/ml, and incubation at 35°C for 72 h (39). Test compound concentrations ranged from 0.1 μg/ml to 49 ng/ml; the MIC was defined as the concentration that prevented any discernible visible growth after 48 h (C. albicans, C. glabrata, and C. parapsilosis) or 72 h (C. neoformans). The MICs for PMB, PME, and octapeptin C4 for A. fumigatus were determined in accordance with the CLSI M38-A2 guidelines (40) using synthetic RPMI 1640 medium (with glutamine, without bicarbonate and with phenol red; Life Technologies, USA). The final inoculum concentration was 0.4 × 10^4^ to 5 × 10^4^ cells/ml, with incubation at 35°C for 48 h. Test compound concentrations ranged from 0.1 μg/ml to 49 ng/ml; the MIC was defined as the concentration that prevented any discernible visible growth after 48 h. All assays were completed in triplicate. Fluconazole was used as a positive control where the MIC was determined at both 50% growth (in accordance with CLSI M27-A2 guidelines) and when there was a “no visible growth” reading.

### *In vitro* time course assay of capsule influence on test compound efficacy.

Capsule induction of strain H99 was achieved as described by Zhai et al. (37) with growth on RPMI 1640 (without l-glutamine and sodium bicarbonate) (Sigma, Australia) agar medium and incubation at 37°C in 5% CO_2_ and capsule suppression by growth on YPD containing 1 M NaCl at 30°C in ambient air. After 3 days, the cells from each culture were collected, washed twice with phosphate-buffered saline (PBS), and resuspended in PBS to avoid changes in the capsule at a density of 1,500 to 2,000 cells/ml. Cell suspensions were treated with test compounds at the determined MIC value. The compounds tested were PMB sulfate, octapeptin C4, and the octapeptin C4 alanine scan derivatives P1L, P1D, P4, P3, P4L, P4D, P5, P6, P7, and P8. At 0, 1, 2, 4, 6, and 8 h after inoculation, samples from each treatment were spread onto a YPD plate, followed by incubation at 30°C for 3 days, at which time the colonies were counted. The assay was repeated in duplicate, and paired sample *t* tests were performed using GraphPad Prism v7.0 (GraphPad Software, USA).

### *In vitro* time course assay of melanization effect on test compound efficacy.

Melanization was induced by growth on l-DOPA agar medium at 30°C and suppressed by growth on YPD agar medium at 30°C. Cells were grown over 3 days and collected, washed twice in PBS, and then resuspended in PBS at a density of 1,500 to 2,000 cells/ml. Aliquots of the cells were treated with PMB sulfate, octapeptin C4, and the octapeptin C4 derivatives P1L, P1D, P4, P3, P4L, P4D, P5, P6, P7, and P8 at the determined MICs. At 0, 1, 2, 4, 6, and 8 h after inoculation, samples of the suspension were spread onto YPD agar and incubated at 30°C for 3 days, and the colonies were counted. The assay was repeated in duplicate, and paired sample *t* tests were performed in GraphPad Prism v7.0.

## Supplementary Material

Supplemental material
