# Supplementary material for: Antimicrobial Octapeptin C4 Analogues Active against Cryptococcus Species
Source: Antimicrob Agents Chemother. 2018 Jan 25;62(2):e00986-17. doi: 10.1128/AAC.00986-17 (PMC5786788; doi:10.1128/AAC.00986-17)
Supplement: Supplemental material [file supp_62_2_e00986-17__index.html]

Supplemental material 

# Antimicrobial Octapeptin C4 Analogues Active against Cryptococcus Species

## Supplemental material

- Supplemental file 1 -

  Table S1 and Fig. S1 to S3

  PDF, 585K
