# Supplementary material for: Antimicrobial Octapeptin C4 Analogues Active against Cryptococcus Species
Source: Antimicrob Agents Chemother. 2018 Jan 25;62(2):e00986-17. doi: 10.1128/AAC.00986-17 (PMC5786788; doi:10.1128/AAC.00986-17)

Supplementary Information
Table S1

		C. neoformans **var.** ***grubii***				
**Strain**	**Ref**	**Origin**	**Fluc MIC (ug/mL)**	**Fluc MIC-2# (ug/mL)**	**Amp B MIC (ug/mL)**	**Oct C4 MIC (ug/mL)**
H99 XL	(1, 2)	lab strain	6.25	3.13	3.13	1.56
H99 stud	(3)	lab strain	6.25	3.13	3.13	1.56
H99O	(3)	lab strain	12.50	6.25	3.13	1.56
486		USA	25.00	12.50	3.13	3.13
488	(4)	AIDS+, pre-HAART USA	25.00	12.50	3.13	1.56
490		USA	12.50	6.25	3.13	1.56
496		USA	12.50	6.25	3.13	1.56
500		USA	12.50	6.50	3.13	1.56
502	(4)	ARR3+ USA	12.50	6.50	3.13	3.13
505		India	6.25	6.25	3.13	1.56
507		India	12.50	6.25	3.13	1.56
510		VNII India	3.13	1.56	3.13	1.56
512		India	12.50	6.25	3.13	3.13
516		India	12.50	6.25	3.13	3.13
518	(5)	VNI India	12.50	6.25	3.13	1.56
522		India	12.50	6.25	3.13	3.13
525		India	25.00	12.50	3.13	1.56
1188	(5)	Japan lung	12.50	3.13	3.13	3.13
1189	(5)	USA BAL/HIV	25.00	12.50	3.13	1.56
1194	(5)	Italy	6.25	1.56	3.13	1.56
1197	(5)	Botswana CSF/AIDS	12.50	3.13	3.13	3.13
1196	(5)	Botswana CSF/AIDS	25.00	12.50	3.13	3.13
1199	(5)	Uganda CSF/HIV+ VNII	12.50	3.13	3.13	1.56

		C. neoformans **var.** ***gattii***				
**Strain**	**Ref**	**Origin**	**Fluc MIC (ug/mL)**	**Fluc MIC-2# (ug/mL)**	**Amp B MIC (ug/mL)**	**Oct C4 MIC (ug/mL)**
R266	(6)	Vancouver Island	12.50	6.25	6.25	1.56
R267	(6)	Vancouver Island	<25	25.00	6.25	12.50
R268	(6)	Vancouver Island	1.56	0.78	6.25	3.13
R269	(6)	Vancouver Island	12.50	3.13	6.25	3.13
R270	(6)	Vancouver Island	6.25	3.13	12.50	3.13
B5742	(6)	VGIV CSF India	6.25	3.13	6.25	3.13
B5748	(6)	VGIV HIV India	12.50	3.13	6.25	3.13
ATCC 24066	(6)	ATCC (NIH 18)	3.13	1.56	6.25	1.56
ATCC 32269	(6)	ATCC	1.56	0.78	3.13	3.13
ATCC 32608	(6)	ATCC (NIH 191)	0.78	0.39	6.25	0.78
ATCC 32609	(6)	ATCC (NIH444)	6.25	3.13	3.13	3.13
ATCC 34878	(6)	ATCC	6.25	3.13	3.13	3.13
ATCC 34882	(6)	ATCC	6.25	1.56	3.13	6.25

93/980	(6)	CSF France	3.13	1.56	3.13	3.13
94/943-6	(6)	CSF Mexico	6.25	1.56	3.13	3.13
94/943-7	(6)	CSF Mexico	12.50	3.13	6.25	6.25
94/943-8	(6)	CSF Mexico	1.56	0.78	3.13	1.56
94/943-10	(6)	CSF Mexico	6.25	1.56	6.25	1.56
96/1120-1	(6)	Brain HIV2 France	25.00	3.13	6.25	3.13
96/1120-2	(6)	CSF HIV2 France	25.00	6.25	12.50	3.13

C. neoformans* var. *neoformans

Strain	Ref	Origin	Fluc MIC (ug/mL)	Fluc MIC-2# (ug/mL)	Amp B MIC (ug/mL)	Oct C4 MIC (ug/mL)
WM 01.127		USA	12.50	6.25	3.13	1.56
WM 02.142		Russia	12.50	6.25	6.25	1.56
WM 02.37		Canada	25.00	6.25	3.13	1.56
WM 02.52		Canada	6.25	3.13	6.25	1.56
WM 04.168		Chile	12.50	6.25	3.13	1.56
WM 04.171		Thailand *	6.25	6.25	3.13	1.56
WM 05.467		Brazil *	12.50	6.25	3.13	1.56
WM 05.515		Peru	6.25	1.56	3.13	1.56
WM 09.109		Italy*	6.25	3.13	3.13	1.56
WM 09.112		USA	12.50	3.13	3.13	1.56
WM 10.119		Australia	6.25	3.13	3.13	1.56
WM 1706		Argentina	12.50	6.25	3.13	1.56
WM 1740		Chile	6.25	3.13	3.13	3.13
WM 2207		USA*	25.00	12.50	3.13	1.56
WM 2242		USA	3.13	1.56	3.13	1.56
WM 2530		USA	6.25	3.13	3.13	3.13
WM 629		Australia	6.25	3.13	3.13	3.13
WM 02.141		Russia	6.25	3.13	3.13	3.13
WM 02.143		Russia	12.50	6.25	3.13	3.13

# MIC-2 is 50% fungal growth. Other MIC values in this table are no visible growth.

* denotes strains isolated from the environment rather than human clinical strain.

1. **Zhai B, Zhou H, Yang L, Zhang J, Jung K, Giam CZ, Xiang X, Lin X.** 2010. J Antimicrob Chemother **65:**931-938.
2. **Zhai B, Lin X.** 2013. Int J Antimicrob Agents **41:**250-254.
3. **Janbon G, Ormerod KL, Paulet D, et al.** 2014. PLoS Genet **10:**e1004261.
4. **Ormerod KL, Morrow CA, Chow EW, Lee IR, Arras SD, Schirra HJ, Cox GM, Fries BC, Fraser JA.** 2013. Comparative genomics of serial isolates of *Cryptococcus neoformans* reveals gene associated with carbon utilization and virulence. G3 (Bethesda) doi:10.1534/g3.113.005660.
5. **Litvintseva AP, Thakur R, Vilgalys R, Mitchell TG.** 2006. Genetics **172:**2223-2238.
6. **Fraser JA, Giles SS, Wenink EC, Geunes-Boyer SG, Wright JR, Diezmann S, Allen A, Stajich JE, Dietrich FS, Perfect JR, Heitman J.** 2005. Nature **437:**1360-1364.

Fig. S1. Purity of synthesized compounds

**Compound**	**% Purity by LC-MS**	
	**ELSD**	**UV (254 nm)**
Octapeptin C4	100	100
P1L	100	100
P1D	100	100
P3	100	100
P4L	100	100
P4D	100	100
P5	100	96
P6	100	100
P7	100	100
P8	100	100

Fig. S.2 *In vitro* time-course assay of *C. neoformans* melanisation effect on compound efficacy using strain H99

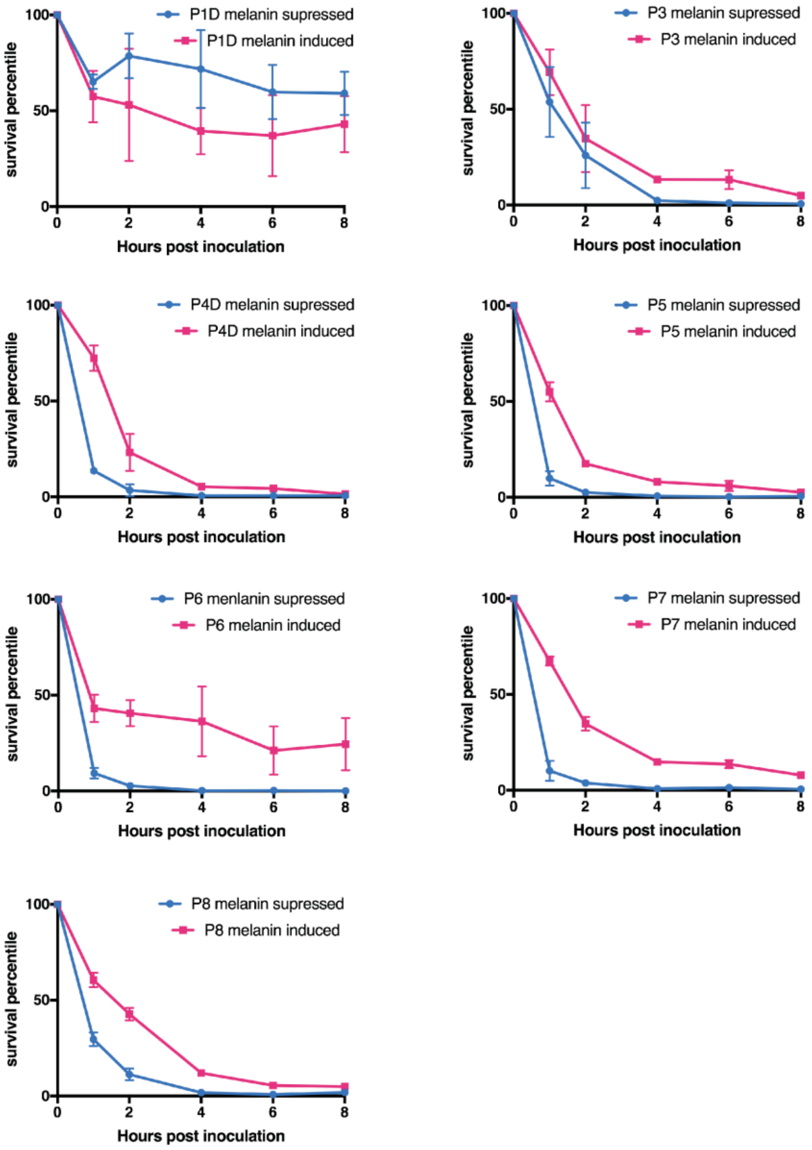

Fig. S.3 *In vitro* time-course assay of *C. neoformans* capsule effect on compound efficacy using strain H99

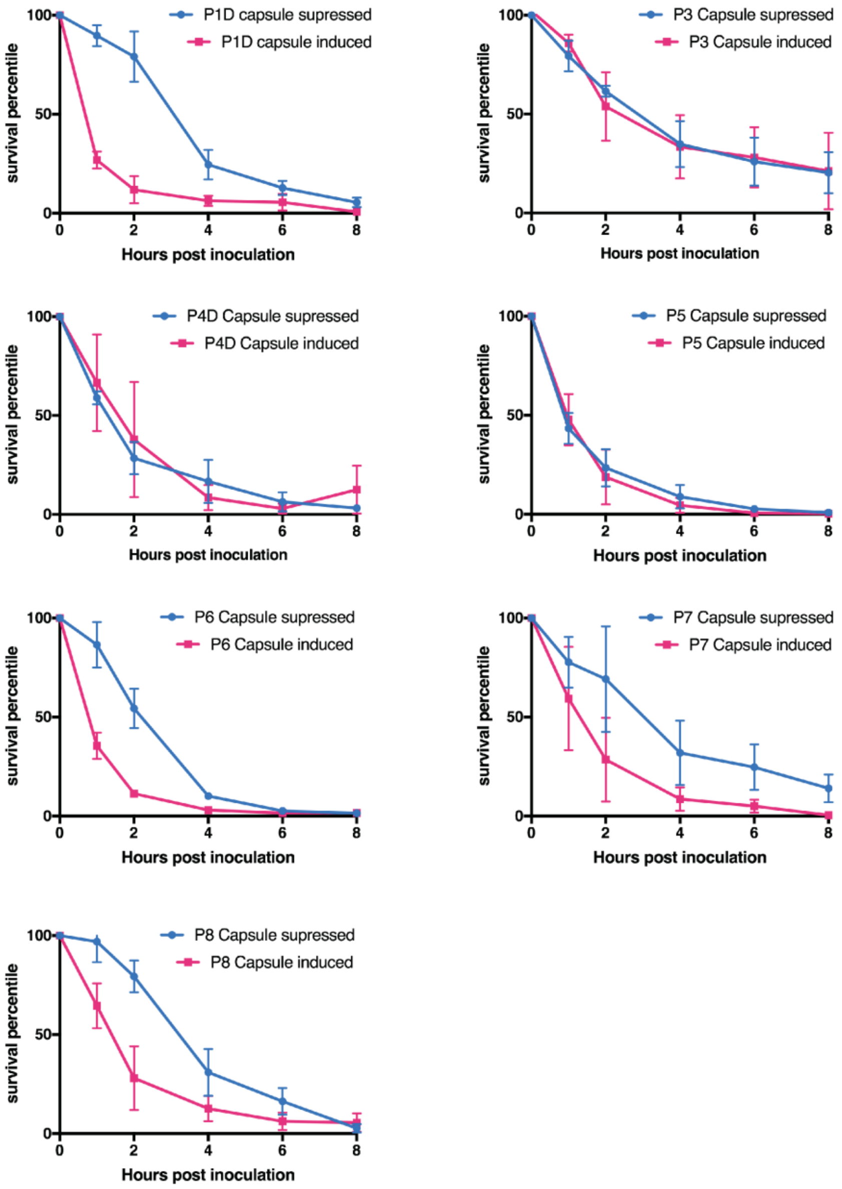

Supplement: Supplemental material [file AAC.00986-17_zac002186874s1.pdf]
